# The Global Transcription Factor FvCon7 Plays a Role in the Morphology, FB1 Toxin Production, and Pathogenesis of *Fusarium verticillioides*

**DOI:** 10.3390/plants14172725

**Published:** 2025-09-01

**Authors:** Gaolong Wen, Xiange Lu, Jiayan Liang, Yi Liu, Xudong Zhang, Guodong Lu, Zonghua Wang, Wenying Yu

**Affiliations:** 1 Fujian Universities Key Laboratory for Plant-Microbe Interaction, College of Life Science, Fujian Agriculture and Forestry University, Fuzhou 350002, China18279801597@163.com (X.L.);; 2 National Key Laboratory of Agricultural and Forestry Biosafety, Fuzhou 350002, China; 3 National Key Laboratory of Agricultural Microbial Resource Exploration and Utilization, Huazhong Agricultural University, Wuhan 430070, China; 4 Key Laboratory of Biopesticide and Chemical Biology, Ministry of Education, Fujian Agriculture and Forestry University, Fuzhou 350002, China

**Keywords:** FvCon7, morphology, conidiogenesis, FB1, cell wall

## Abstract

*Fusarium verticillioides*, an important global pathogenic fungus, compromises crop quality and yield by infecting maize, sugarcane, and some Solanaceae, endangering food security through contaminated grains and cereals with the fumonisin B1 (FB1) toxin. While Con7 has been reported as a transcription factor involved in the sporulation and pathogenicity of some pathogenic fungi, the function of FvCon7 and its regulatory genes in *F. verticillioides* remains uncharacterized. Gene deletion mutants of *ΔFvcon7* were constructed through homologous recombination, which exhibited defects in vegetative growth, survival, sporophore development, conidiation, conidial germination, and carbon metabolism. Carbon metabolism defects led to a significant accumulation of glycogen granules in hypha and lipid bodies in conidia. Additionally, *ΔFvcon7* displayed impaired cell wall structure and integrity, along with an altered expression of genes encoding cell wall-degrading enzymes (such as chitinase), as detected by qRT-PCR. Moreover, Fvcon7 also plays a role in the pathogenicity of maize and sugarcane through different splicing, defective conidia, reduced survival viability, differential expression of secreted proteins, and deficiencies in antioxidant stress capacity. Furthermore, using yeast one-hybrid (Y1H) assays, FvCon7 was found for the first time to directly regulate the expression of *FvFUMs* by binding to the CCAAT box within the promoters of six key *FvFUMs*, thereby affecting FB1 production. Overall, FvCon7 functions as a global transcription factor regulating multiple phenotypes. This study provides a theoretical basis for elucidating the mechanism of transcription factor FvCon7 regulating toxin production and pathogenesis in *F. verticillioides*.

## 1. Introduction

*Fusarium verticillioides* is the predominant pathogen of the genus *Fusarium* in maize and other cereals, causing ear rot in maize and pokkah boeng disease in sugarcane. *F. verticillioides*, a soil-, seed-, and air-borne phytopathogen, can persist in soil or plant residues and infect maize systematically through the roots, seeds, silks, and leaves by external wounds or stomas. *F. verticillioides* not only causes high yield losses, but also contaminates infested grains and cereals with the mycotoxin fumonisn (FB), posing a serious health threat to both humans and livestock [1,2].

Con7 was initially identified as a conidiation protein in *Magnaporthe oryzae* [3]. *CON7* deletion mutants produce abnormal conidia with significantly reduced conidiation in *M. oryzae* [3] in *F. graminearum* [4]. The *CON7* gene is conservative in different species, such as *Colletotrichum graminicola* (*CgCON7*), *C. siamense* (*CsCON7)*, and *M. oryzae (MgCON7)*, containing a typical C_2_H_2_ zinc finger domain and exhibiting nuclear localization [5,6]. The expression of *CON*7 only occurs in the conidia phase, but cannot be detected in the vegetative hyphae of *M. oryzae* [6]. FgCon7 is activated and expressed by the global transcription factor FgHtf1, which promotes the aerial growth and conidial differentiation of *F. graminearum* [7]. Furthermore, *FgCON7* serves as a target gene for FgHtf1, and FgCon7 also binds the promoter region of *FgHTF1* to negatively regulate its expression, thus both forming a negative-feedback loop to mediate sporulation in *F. graminearum* [8].

MgCon7 was shown to be essential for appressorium formation in *M. oryzae* [6,9,10], *C. graminicola*, and *C. siamense* [5]. A *CON7* mutant of *M. oryzae* with an insertion at 1479 bp upstream of the *CON7* gene lead to only 10% of germ tubes forming appressoria [11]. However, the *MgCON7* deletion mutant failed to form appressorium-like structures on hyphal tips, but still produced appressoria by germ tubes on hydrophobic surfaces [12]. In *M. oryzae*, MgCon7 activates the expression of the appressorium formation-related gene chitin-binding protein 1 (CBP1), which is specifically expressed during the early stage of appressorium differentiation [13,14].

*F. oxysporum*-deleting *ΔFocon7-1* exhibited defects in hyphal branching and cell wall structure [15]. *Verticillium* species Con7 ortholog Vta2 is a positive regulator of vegetative growth, H_2_O_2_ detoxification, and virulence [16]. Wheat pathogen *Parastagonospora nodorum* PnCon7 positively regulates Tox3 effector gene expression through the direct binding of a cis-regulatory element (5′-CTCCACCTATCCTAATCTAGTTAAA-3′) and mediating disease [17]. Overall, Con7 serves as a general crucial transcription factor regulating morphogenesis, development, and virulence in plant pathogenic fungi in *M. oryzae* [5,6], *F. oxysporum* [15], *F. graminearum* [4], and *P. nodorum* [17].

Con7 serves as a form of global gene expression. Different species of Con7 regulate different genes to involve diversified pathways. The *CHS7* gene is regulated by MgCon7 in *M. oryzae* [12]; a 29% reduction in the chitin content of germinated spores was observed and the mutant was hypersensitive to the chitin synthase inhibitor nikkomycin Z in the *ΔMgcon7* mutant [6]. The *ΔFgcon7* deletion mutant produces chlamydospore-like structures with high chitin-level accumulation, and the chitin synthase gene *FGSG_06550* shows significant up-regulation in the *ΔFgcon7* deletion mutant [4]. However, the domain of the target recognized by transcription factor Con7 has not been reported. On the other hand, only very few upstream regulatory factors and genes for target Con7 are known [6,7]. Transcription factor FgHtf1 activates FgCon7, which promotes a shift to aerial growth and conidiation in *F. graminearum* [7].

*F. oxysporum* FoCon7 modulates the expression of a large number of genes involved in different biological functions, including host–pathogen interactions, morphogenesis and development, signal perception and transduction, transcriptional regulation, and primary and secondary metabolisms [15]. However, the literature contains no reports of Con7 specifically regulating mycotoxins. Fumonisins are a family of amino-polyalcohols that contaminate cereal-based foods and feeds worldwide and are associated with cancer in rodents. FB1 is a sphinganine analog mycotoxin (SAM) and potent inhibitor of sphinganine N-acyl transferase causing the accumulation of long-chain sphingoid bases and complex sphingolipid depletion. A total of 15 fumonisin synthesis genes are organized in clusters [18]. Among these, the predicted ORF10 and ORF16 proteins share significant similarity to fatty acyl-coenzyme A (CoA) synthetases; ORF12 and ORF15 are highly similar to cytochrome P450 monooxygenases; and ORF17 and ORF18 resemble longevity assurance (LA) factors [19].

In this study, we characterized the Con7 ortholog FvCon7 and identify its potentially regulated genes in the plant pathogenic fungus *F. verticillioides*, which will contribute significantly to our understanding of the genetic pathways that regulate hyphal differentiation, conidiation, and pathogenicity.

## 2. Results

### 2.1. Identification of the C2H2 Zinc Finger Transcription Factor FvCon7 in F. verticillioides

**Figure A1:**
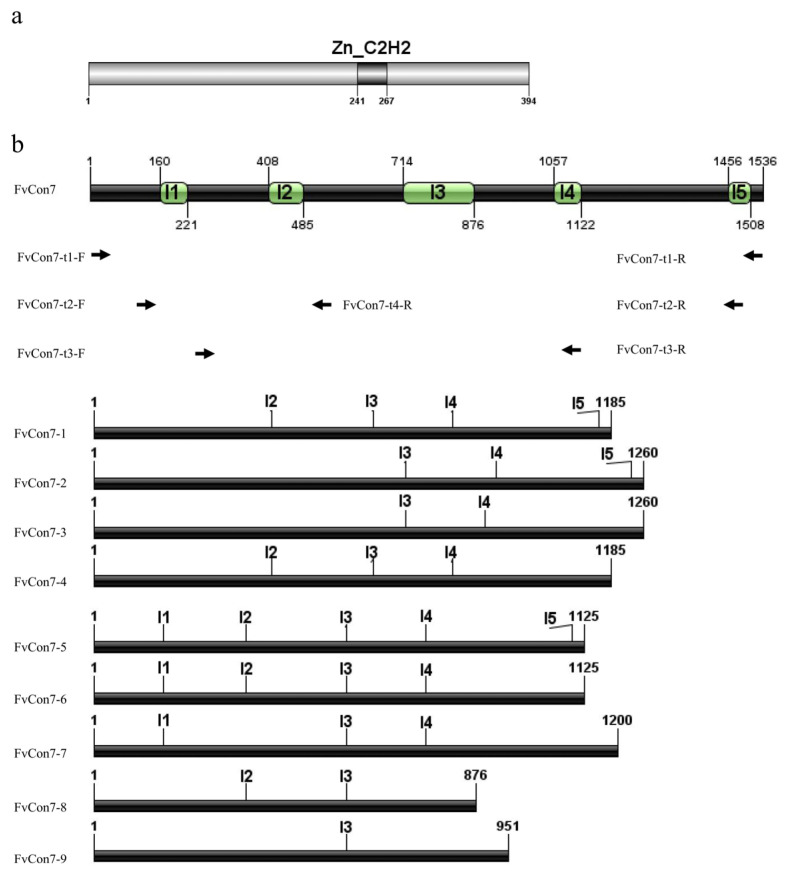

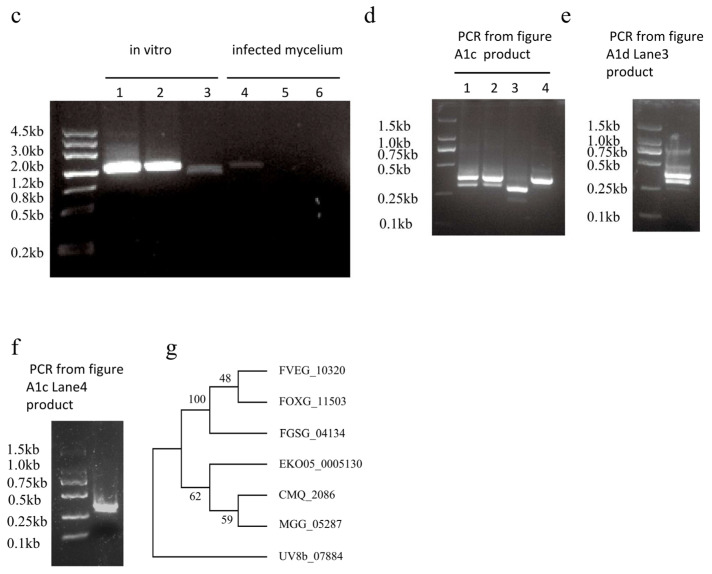
FvCon7 undergoes alternative splicing, generating nine hypothetical protein variants. (**a**) Domain architecture of the transcription factor FvCon7 in *F. verticillioides*. (**b**) Schematic structures of FvCon7-1to FvCon7-9 transcripts, illustrating primer binding sites and splicing patterns. FvCon7-1 retains intron 1 and splices introns 2–5. FvCon7-2 splices introns 3–5. FvCon7-3 splices introns 3 and 4. FvCon7-4 splices introns 2–4. FvCon7-5 splices all five introns (1–5). FvCon7-6 splices introns 1–4. FvCon7-7 splices introns 1, 3, and 4. FvCon7-8 retains introns 1 and 4, splices introns 2 and 3, and terminates early within intron 4. FvCon7-9 retains introns 1, 2, and 4, splices intron 3, and terminates early within intron 4. I1–I5 indicate intron positions from the start codon. Boxed: alternative splicing region. Primer sets used for transcript validation: Pair 1 (FvCon7-t1-F/R) amplifies FvCon7-1, -2, -5; Pair 2 (FvCon7-t1-F/t2-R) amplifies FvCon7-3, -4, -6, -7; Pair 3 (FvCon7-t1-F/t3-R) amplifies FvCon7-8, -9; Pair 4 (FvCon7-t2-F/t4-R) amplifies spans intron I1 and I2 intervals; used to distinguish FvCon7-1 (273 bp), -2 (350 bp), -5 (210 bp); FvCon7-3 (350 bp), -6 (210 bp); and FvCon7-8 (273 bp), -9 (350 bp). t2-F: from intronI1-3 bp to 17 bp, t4-R: from the end 10 bp of intron I2 to outer 10 bp; Primer pair 5: spans I1–I3 including I2; distinguishes FvCon7-4 (180 bp); fragments >250 bp indicate co-amplification of FvCon7-3 and -7. t3-F:intron I1 + 10 bp to + 30 bp. (**c**) Different splicing for FvCon7 transcripts (cDNA products) of mycelia from CM media and infected mycelia by three different primes pares. Lane1,4: Using primers pare 1 to amplify the possible products of FvCon7-1,2,5; Lane2,5: Using primers pare 2 to amplify the possible product of FvCon7-3,4,6,7; Lane3,6: Using primers pare 3 to amplify the possible product of FvCon7-8,9. Results are representative of at least three biological replicates per treatment. (**d**–**f**) Multiple PCR distinguishes the transcription mixture in (**c**). (**d**) Lane 1 (Pair 4, from Lane 1c): >250 bp products → FvCon7-1 (273 bp), -2 (350 bp); no FvCon7-5 (210 bp); Lane 2 (Pair 4, from Lane 2c): >250 bp → FvCon7-3 (350 bp); no FvCon7-6 (210 bp); 273 bp band may correspond to FvCon7-4 or -7; Lane 3 (Pair 4, from Lane 3c): FvCon7-9 (350 bp); no FvCon7-8 (273 bp); Lane 4 (Pair 5, from Lane 2c): FvCon7-4 (180 bp); >250 bp products suggest co-presence of FvCon7-3 and -7; (**e**) Using > 250 bp products from (d) Lane 4 and Pair 4: confirms presence of FvCon7-3 (350 bp) and FvCon7-7 (289 bp); (**f**) Lane 1 (Pair 4, from Lane 4c): >250 bp→FvCon7-1 (273 bp), -2 (350 bp); no FvCon7-5 (210 bp) (**g**) Phylogenetic tree of Con7 homologs in different fungi. Amino acid sequences were aligned using Clustal W, and the MEGA 7.0 software was used to perform phylogenetic analysis using the maximum Likelihood method with 1000 bootstrap replicates. *Fo*, *F. oxysporum*; *Fv*, *F. verticillioides*; *Fg*, *Fusarium graminearum*, *Uv*, *Ustilaginoidea virens*, *Gc*, *Grosmannia clavigera*, *Ar*, *Ascochyta rabiei*.

Based on the FoCon7-1 protein sequence from *F. oxysporum* (FOXG_11503, [15]) and the MoCon7 protein sequence from *M. oryzae* (accession KAI6516264.1, locus MGG_05287.5 [12]), the homologous protein FVEG_10320 from *F. verticillioides* was identified in the NCBI database using BlastP analysis, exhibiting identity values of 98.73% and 67% to FoCon7-1 and MoCon7, respectively. Therefore, FVEG_10320 was designated as FvCon7. Analysis using the SMART website revealed that FvCon7 contains a C2H2-type zinc finger DNA-binding domain (Figure A1a). Furthermore, NLS_Mapper software analysis identified a distinct nuclear localization signal (NLS) (248-GAQQHKRPRRRYE-260) in FvCon7. An examination of the *F. verticillioides* genome in the FungiDB database (https://fungidb.org/fungidb/app, accessed on 24 June 2024) predicted nine transcripts associated with *FvCON7* with five introns (Figure A1b). After combining PCR amplification with different primers (Table A1, Figure A1c) and PCR product sequencing (Figure A1d–f), six corresponding products were successfully amplified from the cDNA derived from mycelia in CM culture conditions, while only one product (FvCon7-2) was derived from infected mycelia (Figure A1d,g).

To further investigate the conservation of the FvCon7 protein across eukaryotes, we retrieved other sequences of FvCon7 homologs from the NCBI database for *F. graminearum* (FGSG_04134), *Ustilaginoidea virens* (Uv8b07884), *Grosmannia clavigera* (CMQ2086), and *Ascochyta rabiei* (EKO05005130). These four retrieved homologs exhibited sequence identities of 89.05%, 76.46%, 83.45%, and 84.15% to FvCon7. Subsequently, these four Con7 homologs, after adding Fvcon7, MgCon7, and FoCon7, were aligned using clustalW and the maximum likelihood method to construct a phylogenetic tree. Phylogenetic analysis revealed that FvCon7, FoCon7, and FgCon7 formed a distinct clade with a higher homology, within which FvCon7 was more closely related to FoCon7, while ArCon7, MgCon7, and GcCon7 clustered in a separate clade, and UvCon7 formed another distinct clade (Figure A1g).

### 2.2. FvCon7 Contributes to a Variety of Morphological Defects in Vegetative Growth and Conidia Development

#### 2.2.1. Generation of ΔFvcon7, ΔFvcon7-C, and FvCON7:GFP

**Figure A2:**
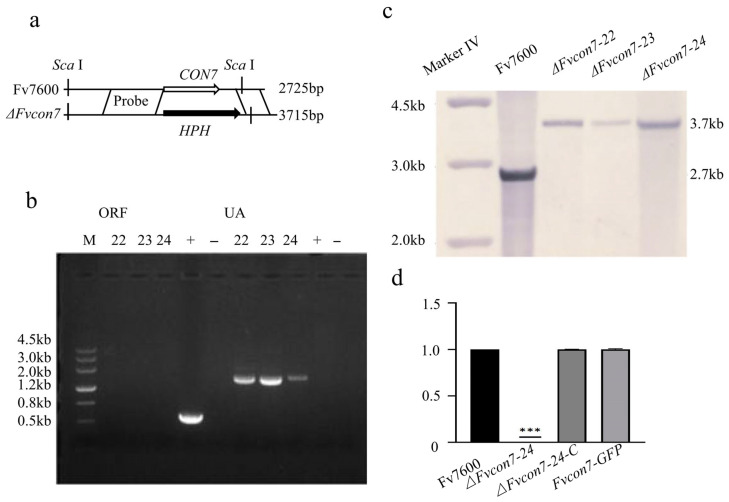
Construction strategy and identification of mutants *ΔFvcon7* and *ΔFvcon7-C.* (**a**) Schematic diagram of gene homologous knockout; (**b**) PCR verification of *ΔFvcon7* mutants; ORF: open reading frame; UA: the connection product of the upstream fragment and the hygromycin fragment; (**c**) Southern blot verification of *ΔFvcon7* mutants with the genome using *Sca* Ⅰ enzyme digestion; (**d**) qRT-PCR verification of *ΔFvcon7* mutants and *ΔFvcon7-C*. Asterisk means significant difference (***, *p* < 0.001, *t*-test).

To assess the function of FvCon7 on both the physiological and pathological outcomes, we generated deletion mutants by replacing *FvCON7* with hygromycin (*HPH*) in the wild-type strain of Fv7600 (WT) (Figure A2a) and confirmed it by PCR assay without an open-reading frame (ORF) and with the connection product (UA) (Figure A2b). Then, *ΔFvcon7* mutants were further confirmed by Southern blot. Following the digestion of genomic DNA with *Sca* I, the fragment sizes hybridized with an upstream probe were about 2.7 kb in the WT and about 3.7 kb in *Δ Fvcon7*, which was consistent with the prediction (Figure A2a,c). Furthermore, we generated a complementation strain (*ΔFvcon7*-C) and a fused strain (FvCON7:GFP) by fusing the *FvCON7* ORF, either with or without a green fluorescent protein-encoding gene (GFP), to its native promoter and introduced the resulting constructs to the *ΔFvcon7* mutant. The successful generation of the *ΔFvcon7*-C and FvCON7:GFP strains was confirmed by qRT-PCR (Figure A2d).

#### 2.2.2. FvCon7 Is Essential for Vegetative Growth

**Figure 1:**
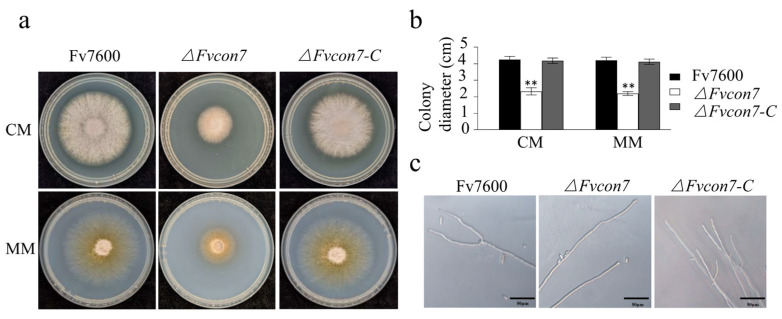
FvCon7 regulated the morphological vegetative mycelium growth of *Fusarium verticillioides*. (**a**) The colony growth of *F. verticillioides 7600* (WT), *ΔFvcon7*, and *ΔFvcon7-C* on the complete medium (CM) and minimum medium (MM) 3 days after culture; (**b**) the growth of the WT as a control and the statistical analysis of colony diameter of the WT, *ΔFvcon7*, and *ΔFvcon7-C*; Asterisk indicates significant difference (**, *p* < 0.01, *t*-test). (**c**) the apical hyphal branches decreased in *ΔFvcon7*. Scale bar = 50 µm.

The wild-type Fv7600 (WT), *ΔFvcon7* mutant, and complemented strain *ΔFvcon7-C* were cultured on CM and MM medium at 25 °C, respectively. The colony size of the *ΔFvcon7* mutant on both the CM and MM was significantly reduced compared with the WT and *ΔFvcon7-C* strains (Figure 1a,b). What is more, most of the apical branches of the WT and *ΔFvcon7-C* were 2-4, while the mutant *ΔFvcon7* had no branch (Figure 1c). These results indicate that the deletion of *FvCON7* affects the mycelial growth rate and the number of apical branches of *F. verticillioides*. Furthermore, *ΔFvcon7* exhibited reduced viability, surviving for only 1.5 years when preserved on dry filter paper at 20 °C, whereas the WT survived for 3–5 years under identical conditions.

#### 2.2.3. FvCon7 Is Essential for Conidia Genesis

**Figure 2:**
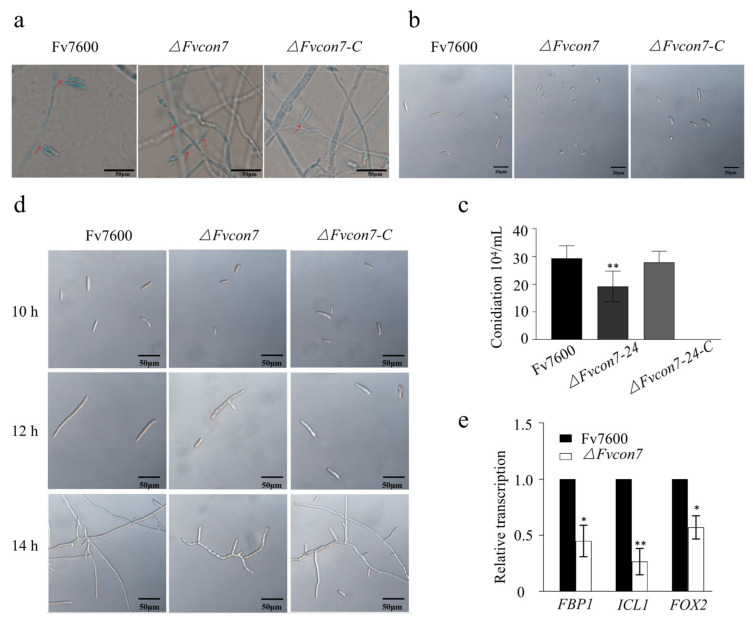
FvCon7 regulated the morphological conidia of *Fusarium verticillioides*. (**a**–**c**) The loss of *FvCON7* affected the spore production, spore morphology, and sporophore length of *F. verticillioides* stained with cotton blue: (**a**) conidiophore formation. Bar = 50 µm; (**b**) spore morphology. Bar = 50 µm; (**c**) spore production. Bar = 20 µm. (**d**,**e**) The loss of *FvCON7* affected conidial germination and the expression of related genes: (**d**) the conidial germination rate of the *ΔFvcon7* mutant decreased significantly. Bar = 50 µm; (**e**) the expression of genes related to spore germination decreased. Error bars denote standard errors of three independent experiments. Asterisk indicates significant difference (*, *p* < 0.05, **, *p* < 0.01, *t*-test).

On the other hand, the morphology of conidiophore and conidia were observed, and the number of conidia were counted in the strains (Figure 2a–c). Compared to WT Fv7600 and *ΔFvcon7-C*, the conidiophore of the *ΔFvcon7* mutant was much longer. The length of the conidiophore of the mutant was approximately twice that of the wild type (Figure 2a). However, the conidia were much smaller. The conidia length of the mutant was approximately one-third that of the wild-type and compensatory strains (Figure 2b). Furthermore, the number of conidia reduced in the *ΔFvcon7* mutant (Figure 2c). The spore germination of strains was observed after 10, 12, and 14 h of incubation at 25 °C. The Fv7600 and *ΔFvcon7-C* spores exhibited polarization and the initiation of germination at 10 h, with a germination rate reaching approximately 50% at 12 h and near 100% at 14 h. However, the spores of *ΔFvcon7* were not germinated at 10 h, and some of the mutant spores displayed polarization and tip sharpening at 12 h. However, after 14 h, the spore germination rate of *ΔFvcon7* was still below 50%, significantly lower than that of Fv7600 and *ΔFvcon7-C* during the same time period (Figure 2d). Consequently, the expression levels of three spore germination-related genes (*FvFBP1*, *FvICL1*, and *FvFOX2*) were assessed by qRT-PCR. These three genes were found to be significantly down-regulated in the *ΔFvcon7* (Figure 2e).

### 2.3. FvCon7 Is Involved in Cell Wall Structure and the Regulation of Different Stresses

**Figure 3:**
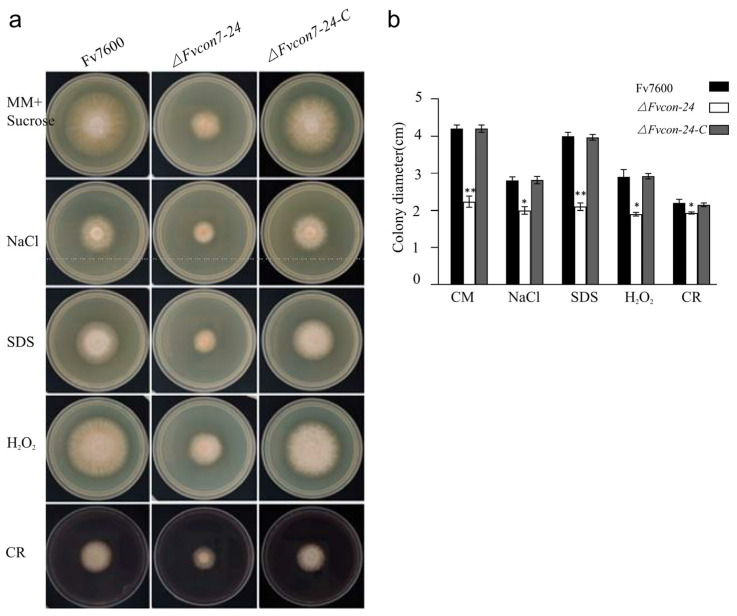
FvCon7 was involved in various stresses. (**a**) The colony growth of the WT, *ΔFvcon7*, and *ΔFvcon7-C* under different stress conditions. The strains were inoculated on MM with the addition of sucrose and further supplemented with various stress inducers (1.0 M NaCl; 0.01% SDS; 0.05% H_2_O_2_ and 200 μg/mL of Congo red, CR for 3 days at 25 °C); (**b**) using the growth inhibition rates of the WT as a control, stress inhibition rates under different stress conditions were analyzed. Error bars denote standard errors of three independent experiments. Asterisk means significant difference (*, *p* < 0.05, **, *p* < 0.01, *t*-test).

**Figure 4:**
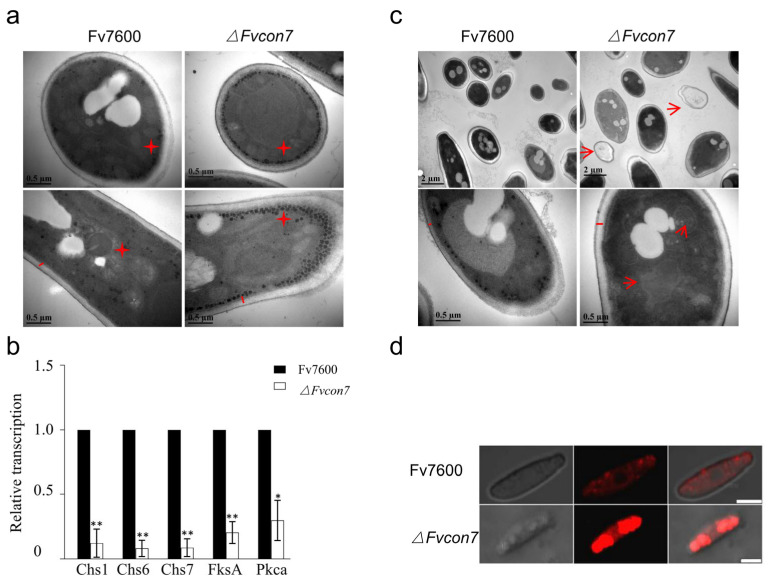
Histological visualization of *ΔFvcon7*. (**a**) A large number of glycogen particles accumulating in an orderly manner along the cell membrane in *Δ Fvcon7* mycelia were observed by transmission electron microscopy (TEM). The left panel is the cross-section of the mycelium, and the right panel is the longitudinal section of the mycelium. Asterisk indicating electron high-density glycogen, bar = 0.5 µm; (**b**) FvCon7 regulated the expression level of cell wall syntheses genes. The relative expression level of the chitin synthase genes CHS1, 6,7, FksA, and Pkca in the *ΔFvcon7* mutant compared to the WT. β-tubulin gene was used as an internal control. Error bars denote standard errors of three independent experiments. Asterisk means significant difference (*, *p* < 0.05, ** *p* < 0.01, *t*-test); (**c**) a large number of low-electron-density granules accumulating in the *ΔFvcon7* conidia were observed by TEM. The left panel is the cross-section of the mycelium, and the right panel is the longitudinal section of the mycelium. Arrow indicates low-electron-density substance; straight line indicates cell wall thickness, bar = 0.5 µm; (**d**) liposomes stained by Nile red inside the *ΔFvcon7* conidia were observed. Bar = 10 µm.

The WT Fv7600, *ΔFvcon7* mutant, and complementing strain *ΔFvcon7*-C were cultured in CM with the addition of the cell wall formation inhibitor Congo red (CR), cell membrane stress factor sodium dodecyl sulfate (SDS), osmotic stress high-concentration NaCl, and oxidative stress H_2_O_2_, respectively, and the growth of *ΔFvcon7* was significantly inhibited under the various stresses presented above (Figure 3a,b). To further assess the ability of resistance to cell wall-degrading enzymes, three-day-old mycelia from CM were treated with the cell wall-damaging agents driselase (0.1 g/10 mL) and lysozyme (0.5 g/10 mL) in lysis buffer. In Fv7600, mycelial cells started collapsing after 90 min of incubation, leading to the production of some protoplasts by 180 min. However, in the same condition, no collapsed cells or protoplasts were observed in *ΔFvcon7*. Compared to Fv7600, *ΔFvcon7* is more resistant to driselase and lysozyme. Consequently, we observed the cell wall’s structure by electron microscope, and found that the cell wall of *ΔFvcon7* was thicker than that of the wild type (Figure 4a). However, the expression levels of five cell wall synthase genes, *FvCHS1* (*FVEG_02839*), *FvCHS6* (*FVEG_07280*), *FvCHS7* (*FVEG_07296*), *FvFSA* (*FVEG_12144*), and *FvPKCA* (FVEG_06268), in *ΔFvcon7* were significantly reduced following detection by qRT-PCR (Figure 4b).

### 2.4. Fvcon7 Is Involved in Carbon Metabolism Regulation

Under the electron microscope, glycogen has a high electron density, with a diameter in the range of 10–40 nm, which can aggregate into chrysanthemum-shaped clusters (50–100 nm). In the *ΔFvcon7* mutant, a high amount of glycogen with diameters ranging from 35 to 90 nm accumulated near the mycelial cell membrane, especially in the tip of the mycelia (Figure 4a). Moreover, under the electron microscope, it was observed that a large number of low-density material vesicles accumulated in the *ΔFvcon7* mutant spores (Figure 4c). Subsequently, we further stained the spores with Nile red and observed a larger area of internal red parts of the liposomes in the *ΔFvcon7* mutant spores, and they were all clustered together, while the wild type only had a weaker red luminescence (Figure 4d).

The excessive accumulation of glycogen reflects the low utilization efficiency of the carbon source, resulting in insufficient energy/carbon skeleton supply for growth, therefore affecting the germination of the spores. The growth of the top of the hypha depends on the directional transport of vesicles (including cell wall synthase, membrane lipids, etc.) to the cell membrane. A large number of glycogen particles (20–100 nm) near the membrane may form a physical barrier, hinder the fusion of vesicles and membrane (similar to a “traffic jam”), and delay the extension of the cell wall, therefore affecting the growth of the colony.

### 2.5. Fvcon7 Is a Transcription Factor of FB1

**Figure 5:**
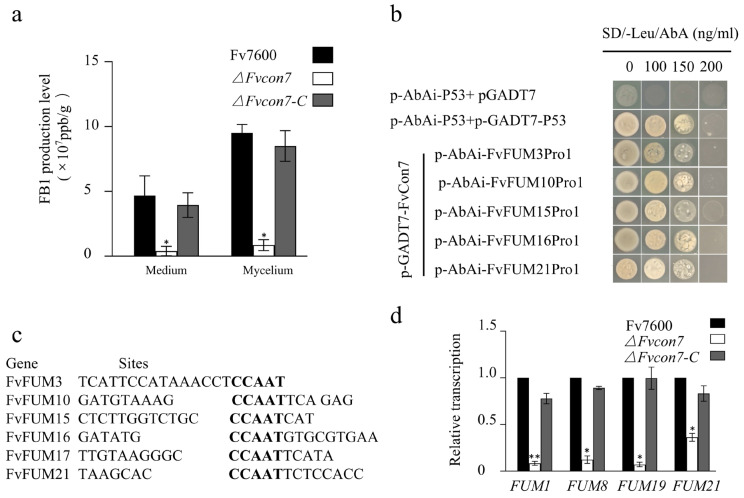
FvCon7 as a transcription factor involved in the FB1 product. (**a**) The FB1 product was quantified and statistically analyzed using the amount of FB1 produced by the WT as the control; (**b**) yeast one-hybrid showing FvCon7’s binding ability to the promoter sequence of *FUM3, 15, 16, 17, 21*; (**c**) FvCon7 can bind the promoters of the six *FUM* genes at the CCAAT site by Multiple EM for Motif Elicitation (MEME 5.4.0) software; (**d**) the relative expression levels of the FB1 biosynthesis-related genes (*FUM*1, 8, 19, 21) in the *ΔFvcon7* mutant were detected and compared to those in the WT. Error bars denote standard errors of three independent experiments. Asterisk means significant difference (*, *p* < 0.05; **, *p* < 0.01, *t*-test).

**Figure A3:**
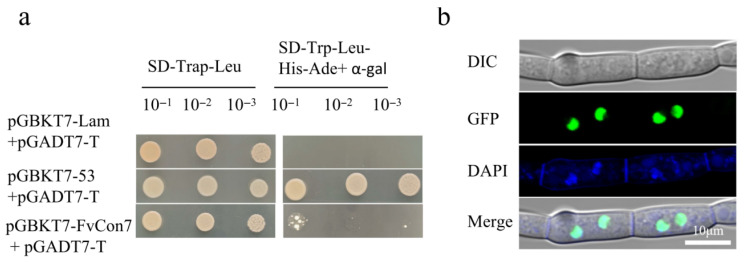
FvCon7 is transcription factor (TF). (**a**) FvCON7-BD were verified to self-activation by yeast-two-hybrid. The co-transformed yeast strain AH109 with *FvCON7* BD vector and pGADT7 vector were initially plated onto SD/-Trp/-Leu (DDO) solid medium for preliminary screening, subsequently transferred to SD/-Trp/-Leu/-His/-Ade (QDO) solid medium for further selection. The results showed that the co-transformed strains grew on the DDO and QDO; (**b**) Cellular localization of FvCon7-GFP fusion proteins. A fusion protein FvCon7:GFP strain was constructed to determine the FvCon7 subcellular localization. Stained with nuclear dye 4′,6-diamidino-2-phenylindole (DAPI), the green fluorescence signal of FvCon7:GFP co-localized with the blue fluorescence of the DAPI, demonstrating that FvCon7 was located in the cell nucleus. Bar = 10 µm.

The *CON7* gene, as a transcription factor (TF), has a transcriptional function in multiple ascomycetes, such as *C. graminicola*, *C. siamense*, and *M. oryzae* [5,6]. We found that FvCON7 in *F. verticillioides* also localized in the nucleus and possessed a transcriptional function (Figure A3). The WT Fv7600, *ΔFvcon7*, and *ΔFvcon7-C* were inoculated onto a solid cornmeal medium and cultured at 25 °C for 10 days. Fumonisin FB1 produced by Fv7600, *ΔFvcon7*, and Δ*Fvcon7-C* in the mycelia and medium was detected, and the results show that FB1 not only accumulated in the mycelia, but also discharged into the culture medium in all strains. Compared with Fv7600 and *ΔFvcon7-C*, the accumulation of fumonisin FB1 in the *ΔFvcon7* mycelium and culture medium was significantly reduced and reached the difference level (Figure 5a).

To elucidate whether FvCon7, functioning as a TF of FB1, directly regulates *FUM*-related genes, 17 *FvFUM* genes related to toxin-producing FB1, including *FvFUM*1, 2, 3, 6, 7, 8, 10, 11, 13, 14, 15, 16, 17, 18, 19, 20, and *FvFUM21*, were all selected as target candidates. First, the software promoter 2.0 was used to predict 17 *FvFUM* genes’ promoter. We randomly designed primers in the predicted promoter region of the *FvFUM* genes. The interaction between FvCon7 and these promoters was investigated by the yeast one-hybrid (Y1H) method. The results show that FvCon7 can bind to the promoters of *FvFUM*3, 10, 15, 16, 17, and 21 (Figure 5b), indicting that six *FUM*-related genes are directly regulated by FvCon7. Further analysis using Multiple EM for Motif Elicitation (MEME) revealed that FvCon7 may bind to the CCAAT cis-regulatory element regions in the promoters of six *FUM* genes (Figure 5c). Based on the ChIP-qPCR results from FgCon7 showing higher enriched binding to CCAAT cis-regulatory elements compared to the non-specific genomic regions [8], we demonstrated the binding ability of FvCon7 to the promoter of these selected target *FUM* genes at this motif.

Based on the fact that a large amount of the FB1 toxin produced by *F. verticillioides* was secreted outside the cell and rapidly decreased in the *ΔFvcon7* mycelium and culture medium, we detected the expression levels of four key genes—*FvFUM* 1, 8, 19, and *FvFUM*21—related to FB1 synthesis or efflux using qRT-PCR. The expression levels of these four genes in *ΔFvcon7* were significantly lower than those in Fv7600 and Δ*Fvcon7-C* (Figure 5d).

### 2.6. FvCon7 Contributes to Virulence

**Figure 6:**
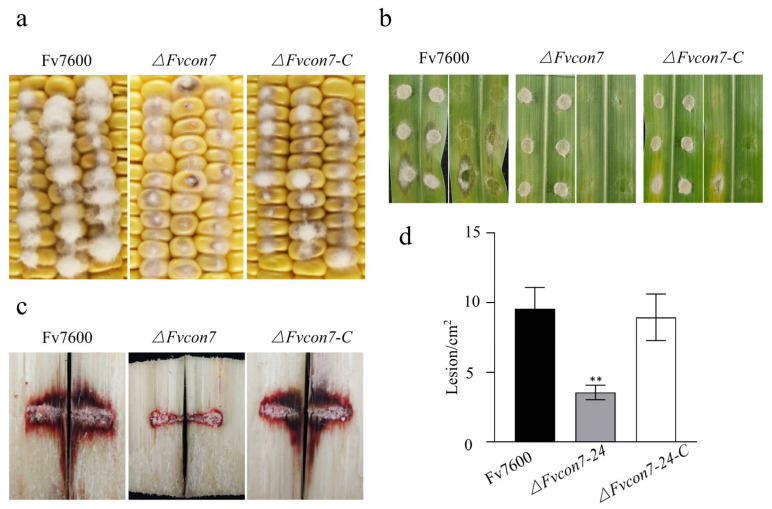
FvCon7 is involved in virulence. Various *Fusarium verticillioides* strains were used, including *ΔFvcon7*, the complementary strains con7-C, and WT Fv7600. (**a**) Virulence assay on maize cob. Kernels of a maize cob (susceptible cultivar B73) were puncture-inoculated using a needle dipped in a conidial suspension and incubated for 3 d; (**b**) virulence assay on leaf. The maize leaves (B73) were inoculated with mycelial discs for 3 d; (**c**) virulence assay on sugarcane (R570) stem. Sugarcane stems were split longitudinally to visually inspect rot symptoms after 7 dpi. The sugarcane stem was inoculated by immersing the conidia tooth tip at the internodal region; (**d**) the area of discoloration of the split longitudinal section of sugarcane was quantified by Image J software and statistically analyzed with the discolored area of the WT infected sugarcane as the control. Error bars denote standard errors of three independent experiments. Asterisk means significant difference (**, *p* < 0.01, *t*-test).

To investigate whether FvCon7 has a pathogenic function, we inoculated various strains into maize kernels, leaves, and sugarcane stems to observe the pathogenic reaction. Compared with Fv7600 and *ΔFvcon7-C*, the pathogenicity in the maize kernels of *ΔFvcon7* was significantly reduced with the mycelium sparse, while the mycelia of Fv7600 and *ΔFvcon7-C* grew abundantly and the maize kernels were rotten and blackened (Figure 6a). Notably, *ΔFvcon7* mutants did not induce any symptoms on maize leaves, whereas Fv7600 exhibited typical symptoms of infection, including a watery texture, chlorosis, and etiolation (Figure 6b). After 10 days of inoculation, the sugarcane stems were split longitudinally from the inoculation site, the size of the lesion was observed, and the area of the lesion was measured with Image J 2.0 software. The area of the lesion was significantly reduced and reached a significant level in the mutant *ΔFvcon7* (Figure 6c,d), indicating that the pathogenicity of the mutant *ΔFvcon7* to sugarcane stems was significantly reduced. This suggested that FvCon7 in the plant pathogenic fungi *F. verticillioides* is involved in pathogenicity.

### 2.7. FvCon 7 Plays an Important Role in Multiple Metabolic Pathways

**Figure 7:**
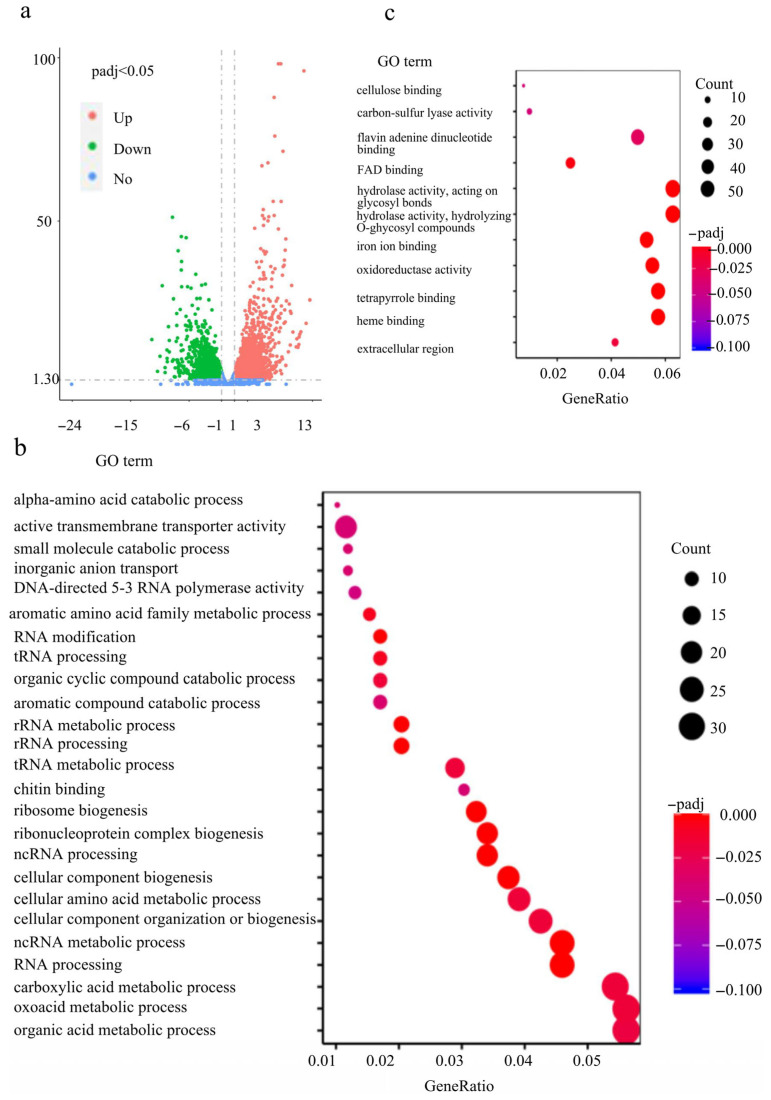

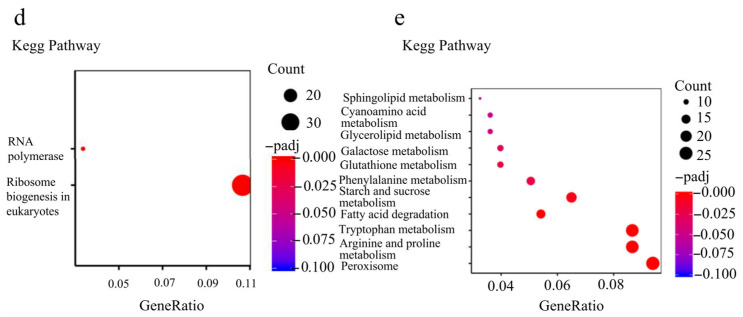
RNA-Seq analysis of the *ΔFvcon7* mutant. (**a**) Volcano maps for differential expression genes (DEGs) identified by |log2_fold change| > 1.2; the fold change was obtained by calculating the ratio of the *ΔFvcon7*/WT to the FKPM values. DEG analysis was conducted using cuffdiff v2.1.1 with the following parameters: FDR (False Discovery Rate) ≤ 0.05, --library–norm-method = classic-fpkm --/-multi-read-correct, and--frag-bias-correct. Red dots: significantly up-regulated genes. Green dots: significantly down-regulated genes. Blue dots: non-differentially expressed genes. (**b**,**d**) The bubble maps for the enriched genes of down-regulated DEGs by GO analysis and KEEG analysis; (**b**) x-axis displays the number of genes in GO terms and the right y-axis shows GO terms; (**d**) x-axis displays the number of genes in the KEEG pathway and the right y-axis shows the KEEG pathway; (**c**,**e**) bubble maps for the enriched genes of up-regulated DEGs by GO analysis rate and KEEG analysis; (**c**) x-axis displays the rate of genes in GO terms and the right y-axis shows GO terms; (**e**) x-axis displays the number of genes in the KEEG pathway and the right y-axis shows the KEEG pathway. The size of the dot indicates the number of genes, and the different colors represent different Padj in (**b**–**e**).

Given FvCon7’s role as a transcription factor involved in regulating growth, plant pathogenicity, and fumonisin B1 (FB1) production, we sought to identify its downstream target genes to elucidate the mechanisms underlying these phenotypes. In order to comprehensively investigate the diverse function of FvCon7, the transcriptome RNA of wild-type Fv7600 and mutant *ΔFvcon7* was sequenced (RNA-seq), and differentially expressed genes (DEGs) were analyzed at the maize kernel-infested stage, as in the study of Peng (2024) [20]. According to the RNA-seq data, there were 2625 up-regulated genes and 1880 down-regulated genes in *ΔFvcon7* compared with Fv7600 (Figure 7a).

We searched for the transcription factors (TFs) for *F. verticillioides* Fv7600 on the FTFD website (http://ftfd.snu.ac.kr, accessed on 24 June 2024). Compared to Fv7600, the mutant *ΔFvcon7* exhibited a down-regulation of 84 TFs and up-regulation of 58 TFs among differentially expressed genes (DEGs), including the down-regulated TF *FvFum21*. The effector proteins of phytopathogenic fungi have the characteristics of low molecular weight (<300 amino acids) and rich cysteine content (>4 cysteine residues) in addition to predicting secretion characteristics or extracellular localization. According to these characteristics, we used signalp, wolf PSORT, tmhmm, gpi-som, and other software to predict the possible secretory proteins in the genome of Fv7600, with a total of 327 secretory proteins being identified. Among the DEGs between Fv7600 and *ΔFvcon7*, 43 secreted proteins increased and 55 decreased in *ΔFvcon7* (e.g., Kp4, major allergen and endo-chitosanase down-regulated).

GO annotation and KEGG enrichment pathways were further analyzed in DEGs [21,22]. The down-regulated DEGs were enriched to 25 GO terms (Figure 7b), and the top 5 down-regulated GO terms were mainly associated with organic acid metabolic process, oxoacid metabolic process, carboxylic acid metabolic process, ncRNA processing, and RNA processing. The 25 GO terms in down-regulated DEGs belong to three levels: biological process level involving RNA synthesis; cellular component level encompassing the plasma membrane, Golgi apparatus, and nucleus; and molecular function level mediating chitin binding, active transmembrane transporter activity, and DNA-directed 5′-3′ RNA polymerase activity. As for the KEGG pathways, the down-regulated DEGs were only enriched to ribosome biogenesis and RNA polymerase (Figure 7d). The up-regulated DEGs enriched 11 GO terms (Figure 7c), and the top 5 up-regulated enrichment pathways were associated with hydrolase activity, acting on glycosyl bonds, hydrolase activity, hydrolyzing O–glycosyl compounds, heme binding, tetrapyrrole binding, and oxidoreductase activity/acting on paired donors/with the incorporation or reduction of molecular oxygen. The 11 GO terms in the up-regulated DEGs belong to three levels: biological process level involving protein phosphorylation, nucleotide metabolic process, and polysaccharide metabolic process; cellular component level encompassing an extracellular region, proton-transporting two-sector ATPase complex, and endoplasmic reticulum membrane; and molecular function level mediating heme binding, oxidoreductase activity, acting on paired donors, with the incorporation of or reduction in molecular oxygen, iron ion binding, and hydrolase activity/hydrolyzing o–glycosyl compounds (Figure 7c). The up-regulated DEGs were enriched to 11 KEGG pathways, and the top 5 DEGs were peroxisome, arginine and proline metabolism, tryptophan metabolism, starch and sucrose metabolism, and fatty acid degradation (Figure 7e). The analysis of enrichment pathways, combining both GO and KEGG enrichment, showed that the deletion of *FvCON7* affects transcription, RNA processing, the cell wall, carbon metabolism, and peroxisome.

## 3. Discussion

Con7 has been reported to have different splicing variants [6,15,17,23]. *M. oryzae CON7* pre-mRNA is processed into different mRNAs, and in the conidial stage, the cDNA splicing variant does not have an additional intron, which was present in a previously reported hypha *CON7* splicing variant [10]. The alternative splicing of the transcription factor gene *CON7* was required for appressorium formation [24]. However, *PnCON7* was confirmed to have four alternative splicing variants during growth in axenic culture and infection by qRT-PCR [17]. PnCon7-1,-3 and PnCon7-4, but not PnCon7-2, specifically interacted with *PnTOX3*. In our study, PCR and sequencing analysis confirmed the presence of six transcript variants under CM culture and of only FvnCon7-2 under infection. We speculate that the six alternative splicing events occurred under different conditions and were associated with distinct downstream target proteins, allowing the organism to adapt to varying environments at different developmental stages. We will further investigate which secreted proteins, particularly those differentially expressed during infection, interact with the transcript FvnCon7-2 to influence pathogenicity, as well as whether other transcripts modulate this interaction to alter pathogenic outcomes.

The *ΔFvcon7* mutants showed impaired growth, which confirmed protein synthesis and carbon metabolism impairments. RNA-seq data further reveal that *FvCON7* deletion disrupts pathways related to ribosome biogenesis and RNA polymerase function. As a result, the mutant *ΔFvcon7* exhibited severely compromised protein synthesis, resulting in dramatically slowed hyphal growth. In addition, a substantial accumulation of glycogen particles was observed adjacent to the apical hyphal cell wall in *ΔFvcon7* mutants. We therefore suggest that the growth of *ΔFvcon7* may be relative to an inability to effectively utilize glycogen. This dysregulation of glycogen catabolism likely causes disruptions in carbon metabolism, resulting in reduced mycelial growth and branching. Moreover, transmission electron microscopy and fat granule staining revealed a significant accumulation of lipid droplets in the cytoplasm of *ΔFvcon7* spores. These droplets appear unable to be mobilized for energy generation, potentially affecting spore germination and further exacerbating defects in carbon metabolism.

Con7 is implicated in maintaining cell wall integrity, enabling the fungus to respond to various stresses by remodeling the cell wall’s structure. Both the mycelia and spores of the *ΔFvcon7* mutant exhibit significantly thickened cell walls, conferring increased resistance to degradation by the cell wall-digesting enzyme driselase and lysozyme compared to wild-type Fv7600. Paradoxically, the mutant shows increased sensitivity to the cell wall biosynthesis inhibitor Congo red (CR). We therefore propose that this discrepancy results from cell wall remodeling in *ΔFvcon7*, involving reduced chitin content and increased glucan levels. Such compensatory adjustments align with the observations of *Saccharomyces cerevisiae ΔScgas1*, where chitin deficiency triggered altered glucan biosynthesis [25], and in *Aspergillus fumigatus*, where nikkomycin Z-induced chitin reduction correlated with β-glucan elevation [26]. Transcriptomic profiling supports this remodeling hypothesis: RNA-seq data reveal a decreased expression of eight chitin-related genes, including chitin synthases and chitin-binding proteins, in *ΔFvcon7*. The down-regulation of *FvCHS1*, *FvCHS6*, and *FvCHS7* was further validated by qRT-PCR. Conversely, GO enrichment analysis revealed an up-regulation of cellulose-binding genes in the *ΔFvcon7* mutant. These findings are consistent with the phenotypes of homologous *CON7* mutants (*ΔFocon7-1*, *ΔMgcon7*, *ΔFgcon7*), in which differentially expressed genes include chitinases, glucanases, lectins, autolysins, and structural proteins [4,6,15]. Notably, regulatory strategies diverge among species: while Con7 directly regulates chitin synthases, such as *MgCHS7* and *FgCHS7*, its ortholog in *F. oxysporum* FoCon7-1 does not regulate chitin synthases, but instead suppresses chitinase/glucanase activity [15]. Interestingly, the *ΔFgcon7* mutant compensates for this through Fg6550-mediated chitin overproduction, forming chlamydospore-like structures despite the down-regulation of *FgCHS7* [4]. These factors indicate distinct, species-specific regulatory mechanisms for chitin synthase complexes. To further substantiate our hypothesis, the direct quantification of chitin, cellulose, and glucan content in *ΔFvcon7* cell walls is essential. Altered cell wall composition likely underlies the mutant’s increased CR sensitivity and osmotolerance to NaCl. Furthermore, *ΔFvcon7* shows elevated susceptibility to membrane stress (SDS) and oxidative stress (H_2_O_2_), suggesting broader defects in stress responses that may affect cellular longevity.

Con7 is conserved in *Ascomycota* and regulates sporulation, though its functional impact varies among species. In *F. graminearum*, conidiation is almost completely abolished in *ΔFgcon7* [8]. In contrast, the mutant of *ΔMgcon7* retains the ability to produce spores but exhibits an abnormal spore morphology and hinders the development of appressorium [6]. In our study, we observed that FvCon7 affected the microspores’ production and its morphology, including external morphology and internal cell wall architecture. In addition, the failure of the *ΔMgcon7* mutant to form appressoria may be partially attributed to its inability to produce sufficient cell wall components or to remodel the cell wall in the correct manner, or to a shortfall in the chitin precursors necessary for conidial cell wall construction after germination [6]. Consistent with these findings, the expression of three key spore germination-related genes in the *ΔFvcon7* mutant was lower than that in the wild type through qRT-PCR.

In this study, we demonstrate that the transcription factor (TF) FvCon7 directly regulates multiple FUM genes by binding the specific CCAAT promoter motif, thereby controlling FB1 biosynthesis. Although CCAAT motifs typically act as positive cis-elements in other systems (e.g., the AnCF complex, *A. nidulans* CCAAT binding factor, in *A. nidulans* regulates *hapB* [27,28], and NRS1-associated CCAAT represses *CBP1* vegetatively [13]), the deletion of the CCAAT motif in NRS1 fails to induce eGFP depression during vegetative growth [13]. Beyond its binding to the *FvFUM21* promoter, FvCon7 directly targets several other *FUM* genes, including those encoding Cytochrome P450 monooxygenases (i.e., *FUM3* and *FUM15*, oxygenating C-5 and C-10 positions, with Fum3 specifically modifying C-5), oxygenating enzymes (*FUM6*, *FUM9*, *FUM12*), fatty acyl-CoA synthetases (*FUM10*, *FUM16*), and a longevity assurance factor (*FUM17*). Thus, FvCon7 acts as a master TF for the *FvFUM* fumonisin synthesis gene cluster. We propose that FvCon7 modulates *F.verticillioides* to produce FB1 by primarily regulating the cluster’s key transcriptional activator, FvFum21, along with FB1 biosynthetic genes. Additionally, non-CCAAT motifs within *FUM* promoters may contribute to the fine-tuning of FB1 regulation. Crucially, FvCon7 and FvFum21 likely co-regulate FB1 biosynthesis, collectively controlling FB1 production in *F. verticillioides*.

Conidia play a critical role in driving disease epidemics in the field. The reduced pathogenicity associated with *CON7* deletion mutants is linked to decreased conidiation, conidia deformity, and incomplete development, all of which can hinder pathogen dispersal, as observed in several plant pathogenic fungi, including *M. oryzae*, *F. oxysporum*, and *F. graminearum* [14]. Similar impairments also appeared in *ΔFvcon7*. In addition, the decrease in the germination rate and shorter survival period of *ΔFvcon7* also contribute to the diminished pathogenicity of *F. verticillioides*. The shorter survival time of *ΔFvcon7* directly affects the amount of initial inoculum available for the subsequent growing season, ultimately affecting the overall accumulation of the pathogen in the field. FoCon7-1’s, but not FoCon7-2’s, function is essential for the full virulence of *F. oxysporum* [15]. In contrast, six transcripts of *FvCON7* have been identified in *F. verticillioides*, with FvCon7-2 being specifically essential for full pathogenicity. Additionally, in *M. oryzae*, MgCon7 acts as a transcription factor necessary for the expression of several genes involved in infection-related morphogenesis [6]. Overall, Fvcon7 plays a role in the pathogenicity of maize and sugarcane through multiple mechanisms, including alternative splicing, defective conidia, reduced survival viability, differential expression of secreted proteins, and compromised antioxidant stress capacity.

FvCon7, a global transcription factor, directly binds to the promoters of both transcription factors and structural genes. Comparative transcriptome analysis revealed that the *ΔFvcon7* mutant exhibited a differential expression of a wide range of genes, including multiple transcription factors and secretory proteins that may function as effectors in various biological pathways. To further identify direct FvCon7-tageted genes, the integration of the ChIP-seq with Y1H will be performed. We will further elucidate the molecular mechanism through which FvCon7 regulates growth, particularly in ribosome biogenesis and glycogen metabolism, as well as its impact on specific transcription factors and effector proteins in *F. verticillioides*. Together, these results demonstrate that FvCon7, functioning as a global TF, orchestrates gene expression involved in aerial hyphal development, conidiophore formation, conidiation, pathogenicity, and FB1 biosynthesis in *F. verticillioides*.

## 4. Materials and Methods

### 4.1. Bioinformatics Analysis

The full-length sequences of the Con7 protein in *F. verticillioides* and other selected homologous species were retrieved from the National Center for Biotechnology Information (NCBI) by using the *F. oxysporum* Con7 protein sequence as a query. Multiple sequence alignment of Con7 homologs was performed using Cluster W 2.1. Subsequently, a phylogenetic tree was constructed with the maximum likelihood method based on the alignment in MEGA 7.0 software (http://www.megasoftware.net, accessed on 25 June 2024), and the reliability of the phylogenetic tree was assessed with bootstrap 1000 replicates [29]. The transcript sequences of the Fvcon7 gene were obtained from the fungiDB 24 (https://fungidb.org/fungidb/app, accessed on 25 June 2024). Protein domains were predicted using the SMART 8.0 online tool (http://smart.emblheidelberg.de/, accessed on 25 June 2024), and the nuclear localization signal was predicted using NLS_Mapper software (http://nls-mapper.iab.keio.ac.jp/cgi-bin/NLS_Mapper_form.cgi, accessed on 25 June 2024).

### 4.2. Strain and Growth Conditions

Knockout mutants were generated in the wild-type strain *F. verticillioides* 7600 (Fv7600), and the complemented strain was constructed in the corresponding mutant background. All strains were cultured on complete medium (CM) or minimum medium (MM) at 25 °C for three days to observe morphology, including colony appearance, spores, and spore pore, as well as to count the spores and measure the colony diameter. For germination assays, spores were inoculated onto liquid CM and incubated at 25 °C with shaking at 180 rpm for 10, 12, and 14 h. To detect intracellular lipid droplets, spores were stained with a Nile red staining kit (C2051S, Beyotime, Shanghai, China) and observed under a confocal microscope (Nikon, Tokyo, Japan).

To detect sensitivity to different stress, the strains were cultured for three days in CM supplemented with the following agents: 0.01% SDS (sodium dodecyl sulfate, for membrane stress), 10 mM of H_2_O_2_ (for oxidative stress), and 100 ug/mL of Congo red (CR, for cell wall stress).

Gene deleted mutants, complementation strains, and GFP-tag strains were constructed, and subcellular localization was observed. Using the primers listed in Table A1, the up- and downstream fragments of the target gene *CON7* were amplified, and the 5′ and 3′ end fragments of hygromycin were synthesized. Then, the upstream fragment was ligated to the 5′ fragment of hygromycin, while the downstream fragment was fused to the 3′ fragment. Then, the two ligated fragments were co-transformed into the WT Fv7600 protoplasts. Transformants were selected from the CM containing hygromycin B. Then, deleted mutants were screened by PCR and further verified by Southern blot analysis as the protocol provided by the manufacturer, using detection kit I (Roche, 11745832910, Shanghai, China). In order to construct complementary strains and GFP-tag strains, the promoter and coding sequences of *FvCON7* were amplified from the genomic DNA of Fv7600. The synthesized fragment was cloned into pKNT to generate the complementation construct, and into the pKNTG vector for the C-terminal GFP fusion construct. The vectors pFvCon7 and pFvCon7-GFP were transformed into the protoplast of the corresponding mutant. Then, the mycelia of the FvCon7-GFP strains were observed by confocal microscope (Nikon, Tokyo, Japan).

### 4.3. Electron Microscope Observation

Samples were collected and pre-fixed as follows: strains cultured in liquid CM for two days were washed with 0.1 M phosphate buffer (pH 7.4). Mycelia and spores were collected separately by centrifugation at 2000 rpm for 5 min. The pellets were fixed with an electron microscope fixative (BL911A, Biosharp, Hefei, China) containing 2.5% glutaraldehyde and 0.1 M of phosphate buffer (pH 7.0–7.5) at 4 °C—for 12 h for mycelia and 24 h for spores. Following pre-fixation, subsequent post-fixation steps, including dehydration, infiltration, embedding, slicing, and staining, were carried out according to the established methods [15]. Finally, the samples are observed using a Philips CM 10 transmission electron microscope.

### 4.4. Yeast Two-Hybrid (Y2H) Assay and Yeast One-Hybrid (Y1H) Assay

Self-activation assays were conducted following our previous protocol [30]. The coding sequence of *FvCON7* was amplified by PCR using the primers listed in Table A1, and then cloned into the pGBKT7 vector to generate pBD-FvCON7. The vector pairs pBD-FvCon7 and pGADT7, pGADT7-T and pGBKT7-lam (negative control), as well as pGADT7-T and pGBKT7-53 (positive control) were co-transformed into *S. cerevisiae* AH109. The growth of the transformant was performed by the SD/-Leu-Trp-His-Ade medium.

For yeast one-hybrid (Y1H) assays, the cDNA of transcription factor *CON7* was cloned into a pGADT7 plasmid to obtain pGADT7-FvCON7, while the promoter of *FUM1* to be tested was inserted into the pAbAi plasmid to obtain *FUM1*pro:pAbAi. Subsequently, both recombinant plasmids were co-transformed into the yeast Y1H-Gold strain. When cultured on the SD/-Leu medium with AbA-resistant plasmid at 30 °C for 2–3 d, the growth of the co-transformed strains was detected to evaluate interactions. The same procedure was performed to test the interaction between FvCon7 and the promoters of an additional 16 *FUM* genes associated with FB1 biosynthesis, namely *FvFUM*2, 3, 6, 7, 8, 10, 11, 13, 14, 15, 16, 17, 18, 19, 20, and *FvFUM21*.

### 4.5. Determination of Pathogenicity and Fumonisin FB1 Production

The spore suspension was adjusted to a concentration of 1 × 10^6^ spores/mL. Inoculations were performed as follows. For maize cob inoculation, kernels of the susceptible cultivar B73 were puncture-inoculated to a depth of 0.1 cm using a needle dipped in a conidial suspension, with 30 kernels inoculated per cob. For maize leaf inoculation, mycelial discs harvested from cultures grown on complete medium (CM) for three days at 25 °C were used. For sugarcane stem inoculation, toothpicks were immersed in the spore suspension for 10 min. A hole approximately 1 cm deep was made in the internode of a susceptible sugarcane cultivar R570 using a surface-sterilized needle. The inoculated toothpick was then inserted into the hole, and the wound was wrapped with sealing film. Fv7600 and mutants were inoculated on maize cob with spores for 3 days, 3-leaf maize leaves with a mycelium disc for 3 days, and sugarcane stems with spores for 7 days. All inoculations were carried out at 25 °C. After incubation, the maize cob, maize leaves, and longitudinally split sugarcane stems along the infection site were photographed according to the previous method [31].

Fv7600, mutants, and supplementary strains were inoculated onto the solid maize powder medium, at 25 °C for 10 days, and fumonisins FB1 was extracted from the mycelia and medium. The FB1 enzyme-linked immunosorbent assay kit (Shenzhen Fende Biotechnology, Shenzhen, China) was used to detect the content of fumonisin according to the previous protocol [20].

### 4.6. RNA Sequencing and Fluorescent Real-Time Quantitative PCR (qRT-PCR) Analysis

As described by [20], surface sterilize maize kernels were inoculated with a 5 uL spore solution per kernel (1 × 10^6^ spores/mL) for 10 days at 25 °C; then, the infected maize kernels were collected to extract the total RNA for sequencing.

*Fv*7600 was cultured at 25 °C for two days, and maize leaves were inoculated with Fv7600 for three days. Total RNA from the conidia or mycelia of *Fv*7600 or *Fv*7600-infected maize leaves was extracted. Subsequently, cDNA was synthesized with the reverse transcription kit (Vazyme, Nanjing, China). RT-PCR analysis was performed with the SuperReal PreMix (SYBR Green) kit (Takara, Japan), with the actin gene (FVEG_02048, a skeleton protein of *F. verticillioides*) as an internal reference. The relative quantification of transcripts was calculated by the 2^−ΔΔCT^ method, with three biological repeats performed for each gene.

## Data Availability

All other relevant data are in this paper.

## Appendix B

**Table A1:** The primers used in this study.

Name	Sequence (5′→3′)	Experimental Use
FvCon7-AF	ATTTCTTGCCTGTGAGCC	Gene knock-out
FvCon7-AR	TTGACCTCCACTAGCTCCAGCCAAGCCGCAGTAGTAGCGCCGTTT	
FvCon7-BF	GAATAGAGTAGATGCCGACCGCGGGTTAAGCCACTAAGACGGATGG	
FvCon7-BR	CTTGTGACCCGACAGACC	
FvCon7-ORFF	GGCGTCTATCCTCAGTCAGC	
FvCon7-ORFR	CCGAGGTCGTTTATGCTGTT	
FvCon7-UA	GGGACTCGTCGGTAGAAC	
YG/F	GATGTAGGAGGGCGTGGATATGTCCT	
HY/R	GTATTGACCGATTCCTTGCGGTCCGAA	
HYG/F	GGCTTGGCTGGAGCTAGTGGAGGTCAA	
HYG/R	AACCCGCGGTCGGCATCTACTCTATTC	
H853	GACAGACGTCGCGGTGAGTT	
FvCon7-PF	GGGTACCGGGCCCCCCCTCGAGTTGTCCTCTGAGTGGACCTT	
FvCon7-OR GFP	TCCTCGCCCTTGCTCACCAT AGTCACCCTCTGCCTCTGCG	
GFPF	ATGGTGAGCAAGGGCGAGGA	
GFPR	CGACCTGCAGGCATGCAAGCTTTTACTTGTACAGCTCGTCCATGC	
FvCon7-ADF	GACGTACCAGATTACGCTCAT ATGTCTCTGGTGCCAACACAG	Yeast-two-hybrid assay
FvCon7-ADR	TATCGATGCCCACCCGGGTGGAATTAGTGGCTTGGGGGTTGAC	
FvCon7-BDF	CTGATCTCAGAGGAGGACCTGCATATGTCTCTGGTGCCAACACAG	
FvCon7-BDR	CGCTGCAGGTCGACGGATCCCCGGGAATTAGTGGCTTGGGGGTTGAC	
FvFUM3-ProF	AAGCTTGAATTCGAGCTTCATTCCATAAACCTCCAAT	Yeast-one-hybrid assay
FvFUM3-ProR	CCTCGAGGTCGACAGATCCCCGCAGGATTGCCAAGATTACT	
FvFUM3-ProF1	AAGCTTGAATTCGAGCTCATGCGTGAGGGTATCCATT	
FvFUM3-ProR1	CCTCGAGGTCGACAGATCCCCGATAAGCAAACTCAACGTTT	
FvFUM10-ProF	AAGCTTGAATTCGAGCTGTATTTAGATTCCGTTATAT	
FvFUM10-ProR	CCTCGAGGTCGACAGATCCCCCTCTGAATTGGCTTTACATC	
FvFUM15-ProF	AAGCTTGAATTCGAGCTGGTGTTTGACTAGCCGAGCT	
FvFUM15-ProR	CCTCGAGGTCGACAGATCCCCAAGGCTTTGAGCAAGGTCAA	
FvFUM15-ProF1	AAGCTTGAATTCGAGCTATAGGGTATGTAGCCACAAC	
FvFUM15-ProR1	CCTCGAGGTCGACAGATCCCCAAGTAACTTGCAACCGATGC	
FvFUM17-ProF	AAGCTTGAATTCGAGCTGCTAGAGAGGCCCAACTTGC	
FvFUM17-ProR	CCTCGAGGTCGACAGATCCCCGCGCTAAATTGTACCCTGAT	
FvFUM16-ProF	AAGCTTGAATTCGAGCTAGAAAGCTGGTGTCTGGAGG	
FvFUM16-ProR	CCTCGAGGTCGACAGATCCCCCCTCAGCTACCGGTTTCATT	
FvFUM16-ProF1	AAGCTTGAATTCGAGCTTAAATCCAGGCAATGACACA	
FvFUM16-ProR1	CCTCGAGGTCGACAGATCCCCACATTATCACCATTACACTC	
FvActin-QF	GTCTGGATCGGTGGTTCTATTC	RT-qPCR
FvActin-QR	ACTTGCGGTGAACGATTGA	
FvFum1-QF	GCTCTAGAGAACCGCACTATTC	
FvFum1-QR	TCAACTGGTACCGCCATATTC	
FvFum8-QF	ATCACCGCCACTGTCTTTAC	
FvFum8-QR	GAAGCGTCGGACTTGATACTT	
FvFum19-QF	CATCACACGAGCACCACTATAC	
FvFum19-QR	GCTGACGAGATGTCCGTAAATAG	
FvFum21-QF	TTGCGAGGATCTGTTCTTCTATC	
FvFum21-QR	TATTACCGAGCTTGCGCTATAC	
FvflbB-QF	GATGGCGATACGAACCAGAA	
FvflbB-QR	CCCAAGACATGATAGGAGTTACC	
FvfluG-QF	GGGTAGTCATTGGGAGATCAAG	
FvfluG-QR	GCCCTTTGTACCCGCTAATA	
FvstuA-QR	TCGAAGTTGCCTGCAGAAT	
FvflbC-QF	GGTATGTTGGCTATGAGTCAGG	
FvflbC-QR	GTCGAATGGGTAGAAGGGAATC	
FvChs1-QF	CTCGGATGGGGTGAACCTG	
FvChs1-QR	GAAACCGGCAAAACGAACGA	
FvChs6-QF	CCCAACGATCGACGAATTGC	
FvChs6-QR	CAAAAACTCGCCGAAAGGCA	
FvChs7-QF	AGCTAGGAAGCGGTACCAGA	
FvChs7-QR	CCAGGGACCGATATGTCTGC	
FvPkcA-QF	TCACAATTCCCCATGAGCCC	
FvPkcA-QR	GGACCGGTCTCTGTTCCTTG	
FvFksA-QF	AATTGTCCTGGCCCTGATGG	
FvFksA-QR	GTTACGCCAAGGTGTCCAGA	
FvCon7-t1-F	ATGTCTCTGGTGCCAACACA	Transcript identification
FvCon7-t1-R	TTAGTGGCTTGGGGGTTG	
FvCon7-t2-R	CTAAGTCACCCTCTGCCTCTG	
FvCon7-t3-R	TTAGGCATCTGTGCTCATC	
FvCon7-t4-F	GTGCTACGGAGTACTCGC	
FvCon7-t4-R	CGAAGCGTAAGTTGGGCT	
FvCon7-t5-F	ATCTGCTGCGCAATATCCT	
